# Oral and intravenous transmission of α-synuclein fibrils to mice

**DOI:** 10.1007/s00401-019-02037-5

**Published:** 2019-06-22

**Authors:** Stephanie Lohmann, Maria E. Bernis, Babila J. Tachu, Alexandra Ziemski, Jessica Grigoletto, Gültekin Tamgüney

**Affiliations:** 1 German Center for Neurodegenerative Diseases (DZNE), Venusberg-Campus 1, Gebäude 99, 53127 Bonn, Germany; 2 Structural Biochemistry (ICS-6), Institute of Complex Systems, Forschungszentrum Jülich GmbH, Wilhelm-Johnen-Straße, 52425 Jülich, Germany; 3 Institut für Physikalische Biologie, Heinrich-Heine-Universität Düsseldorf, Universitätsstraße 1, 40225 Düsseldorf, Germany

**Keywords:** Infection, Parkinson’s disease, Peripheral, Synucleinopathy

## Abstract

Parkinson’s disease and related disorders are neuropathologically characterized by cellular deposits of misfolded and aggregated α-synuclein in the CNS. Disease-associated α-synuclein adopts a conformation that causes it to form oligomers and fibrils, which have reduced solubility, become hyperphosphorylated, and contribute to the spatiotemporal spreading of pathology in the CNS. The infectious properties of disease-associated α-synuclein, e.g., by which peripheral route and with which efficiency it can be transmitted, are not fully understood. Here, we investigated the potential of α-synuclein fibrils to induce neurological disease in TgM83^+/−^ mice expressing the A53T mutant of human α-synuclein after oral or intravenous challenge and compared it to intraperitoneal and intracerebral challenge. Oral challenge with 50 µg of α-synuclein fibrils caused neurological disease in two out of eight mice in 220 days and 350 days, and challenge with 500 µg in four out of eight mice in 384 ± 131 days, respectively. Intravenous challenge with 50 µg of α-synuclein fibrils led to disease in 208 ± 20 days in 10 out of 10 mice and was in duration comparable to intraperitoneal challenge with 50 µg of α-synuclein fibrils, which caused disease in 10 out of 10 mice in 202 ± 35 days. Ten out of 10 mice that were each intracerebrally challenged with 10 µg or 50 µg of α-synuclein fibrils developed disease in 156 ± 20 days and 133 ± 4 days, respectively. The CNS of diseased mice displayed aggregates of sarkosyl-insoluble and phosphorylated α-synuclein, which colocalized with ubiquitin and p62 and were accompanied by gliosis indicative of neuroinflammation. In contrast, none of the control mice that were challenged with bovine serum albumin via the same routes developed any neurological disease or neuropathology. These findings are important, because they show that α-synuclein fibrils can neuroinvade the CNS after a single oral or intravenous challenge and cause neuropathology and disease.

**Electronic supplementary material:**

The online version of this article (10.1007/s00401-019-02037-5) contains supplementary material, which is available to authorized users.

## Introduction

α-Synuclein is a soluble, cytosolic protein with a yet not fully identified function and the ability to adopt one or more pathological conformations that have been identified in cellular inclusions in a group of neurodegenerative conditions designated as synucleinopathies [11, 50, 51, 59]. Most commonly associated with these diseases are Parkinson’s disease (PD), dementia with Lewy bodies (DLB), and multiple system atrophy (MSA). What causes α-synuclein to adopt a pathological conformation in these diseases is largely unknown. While a number of familial mutations can cause PD, including missense mutations in α-synuclein and those resulting in elevated protein levels of wild-type α-synuclein, synucleinopathies are mostly regarded as sporadic diseases [1, 12, 25, 27, 29, 35, 37, 39, 48, 65]. A growing body of evidence shows that in an aggregated state, α-synuclein has prion-like properties. “Classical” prions are infectious conformers of the prion protein (PrP) which cause transmissible spongiform encephalopathies, such as scrapie in sheep, chronic wasting disease in cervids, bovine spongiform encephalopathy (BSE) in cattle, or Creutzfeldt–Jakob disease (CJD) in humans [13, 26, 40]. Dependent on the prion strain and the host, PrP prions can cause CNS disease after natural or accidental horizontal transmission via several entryways, including the intracerebral, intraperitoneal, intravenous, or oral route [32]. Reminiscent of PrP prions, intracerebral challenge of different animal models with synthetic or patient-derived pathological α-synuclein has revealed that CNS pathology propagates in a stereotypic manner by interneuronal transmission [7, 31, 60]. Moreover, distinct strains of pathological α-synuclein are associated with specific diseases, and strain characteristics persist upon repeated passaging in one or between different animal models [62, 64]. Recently, we showed that, similar to what has long been known for prions, intraperitoneal and intraglossal challenge of Tg(M83^+/−^:*Gfap*-luc^+/−^) mice, which express the A53T mutant of human α-synuclein and luciferase, with α-synuclein fibrils results in neuroinflammation and CNS disease after neuroinvasion of pathological α-synuclein from the periphery [4, 5, 10, 17, 22, 24]. Similar to our observations, challenge of TgM83^+/−^ mice with α-synuclein fibrils via intrasciatic, intraperitoneal, or intramuscular injection, or with brain homogenate of patients with multiple system atrophy (MSA) via intraglossal, intraperitoneal, or intramuscular injection was reported to result in neurological disease [2, 3, 17, 44, 45, 63]. Because prions can also be transmitted orally or by blood, for instance, bovine spongiform encephalopathy (BSE) prions have been transmitted from infected cattle to humans by the consumption of tainted beef products, and the resulting variant Creutzfeldt–Jakob disease (CJD) prions between humans by blood transfusion, we wondered whether α-synuclein prions could be transmitted via these two routes as well [20, 21]. To address this question, we challenged TgM83^+/−^ mice expressing the A53T mutant of human α-synuclein with recombinant fibrils of human, wild-type α-synuclein, or bovine serum albumin (BSA) as a negative control by the oral and intravenous route [17]. Whereas none of the BSA-injected control mice developed signs of disease or neuropathology, all of the mice challenged intravenously and up to 50% of the mice challenged orally with α-synuclein fibrils developed neurological disease with α-synuclein pathology in their CNS. Our results unmistakably show that a single challenge with α-synuclein fibrils by the oral, intravenous, or intraperitoneal route can be sufficient for α-synuclein fibrils to invade the CNS and to cause neuropathology and disease in TgM83^+/−^ mice.

## Materials and methods

### Mouse husbandry and inoculations

All studies involving animals were approved by the animal protection committee of the North Rhine-Westphalia State Environment Agency (LANUV). All mice were housed under environmentally controlled standard conditions with a 12 h light/dark cycle and free access to food and water. B6;C3-Tg(*Prnp*-SNCA*A53T)83Vle/J mice (short: TgM83^+/−^ mice, The Jackson Laboratory) were crossed to wild-type C57BL/6 J mice and their progeny genotyped by real-time PCR for the presence of transgenes encoding human α-synuclein with the familial A53T mutation [17]. For challenge with α-synuclein fibrils or bovine serum albumin (BSA), we used 6–8-week-old male and female TgM83^+/−^ mice. Oral challenge was done by way of oral gavage, intravenous and intraperitoneal challenges by injection with a 29-gauge disposable hypodermic needle into the tail vain or peritoneum with 50 µg of sonicated α-synuclein fibrils in 12 µL phosphate-buffered saline (PBS, Sigma) or 50 µg BSA in 25 µL 0.9% (w/v) saline solution (Pierce). A high-dose oral challenge was performed with 500 µg of α-synuclein fibrils in 120 µL or 500 µg BSA in 250 µL. For intracerebral challenge, TgM83^+/−^ mice were anaesthetized with isoflurane and stereotactically injected with 10 µg (2.3 µL) or 50 µg (12 µL) of sonicated α-synuclein fibrils or 50 µg BSA (25 µL) into the right striatum (coordinates: + 0.2 mm relative to the bregma, + 2.0 mm relative to the midline, and 2.6 mm below the dura). For stereotactic delivery, we used a rate of 1.4 µL/min, whereafter we left the syringe in place for five additional minutes before slowly retracting the needle. Challenged animals were monitored daily for health and three times weekly for signs of neurological disease, such as reduced grooming, ataxia, tremor, bradykinesia, akinesia, lethargy, circling, tail rigidity, paraparesis, paralysis, kyphosis, and more. Diseased mice were narcotized with ketamine/xylazine and then transcardially perfused with 0.9% (w/v) saline followed by 10% (v/v) formalin neutral buffer solution (Sigma). Dissected brain and spinal cord samples were fixed overnight with 10% formalin neutral buffer solution for immunohistochemistry. Alternatively, diseased mice were killed and their brains and spinal cords snap-frozen on dry ice and stored at − 80 °C for biochemical analysis.

### Preparation of recombinant α-synuclein protein fibrils

Protein fibrils made of human wild-type α-synuclein were prepared as previously described [10]. *Escherichia coli* cells of the strain BL21(DE3) harboring a pET-3a expression plasmid (Novagen) for α-synuclein were grown at 37 °C in 1 L lysogeny broth containing 0.5 g/L NaCl, ampicillin, chloramphenicol, and 1% (v/v) glucose to an optical density of 0.5 at 600 nm. Protein expression was induced with 0.1 mM isopropyl β-D-thiogalactopyranoside and the cells were grown for additional 5 h at 37 °C. For osmotic shock release of periplasmatic material into the buffer, the cells were pelleted by centrifugation at 6000×*g* for 15 min, and resuspended in 35% sucrose solution in 2 mM EDTA and 30 mM Tris–HCl (pH 7.2), and incubated with shaking at room temperature for 15 min. After a second harvest, the cells were resuspended in 90 mL ice-cold water followed by the addition of 37.5 µL of saturated MgCl_2_. The periplasmatic material was boiled for 20 min and then centrifuged at 4 °C and 5000×*g* for 30 min. For fractional ammonium sulfate precipitation, (NH_4_)_2_SO_4_ crystals were added over a 10-min period to the supernatant (19.4 g/100 mL) to achieve 35% saturation with gentle stirring on ice, after which the centrifugation was repeated. To increase the concentration from 35% to 55% saturation, additional (NH_4_)_2_SO_4_ crystals (11.8 g/100 mL) were added over a 10-min period with gentle stirring on ice, after which the centrifugation was repeated. The pellet was resuspended in 10 mL water and dialyzed three times for 3 h against 20 mM Tris–HCl (pH 8.0). α-Synuclein was purified from the supernatant by Resource Q anion exchange chromatography using 20 mM Tris–HCl (pH 8.0) as binding buffer and 500 mM NaCl in 10 mM Tris–HCl (pH 8.0) as elution buffer on an ÄKTA pure chromatography system (GE Healthcare). α-Synuclein was released from the column using a 30 mL linearly increasing gradient from the binding buffer towards the elution buffer, and dialyzed against 150 mM NaCl in 20 mM Tris–HCl (pH 7.2). α-Synuclein fibrils were assembled in an orbital thermomixer (Eppendorf) by agitation at 900 rpm and 37 °C for 7 days. Fibrils were diluted in PBS to 4.25 μg/μL and sonicated on ice for 1 min with 40 pulses of 0.5 s using a Sonoplus mini20 sonicator (Bandelin). The ToxinSensor Chromogenic LAL Endotoxin Assay Kit (Genscript) was used according to the manufacturers instructions to verify that the endotoxin levels in our fibril preparations were low (> 0.01 EU/mL).

### Atomic force microscopy

Atomic force microscopy was used to evaluate the length distribution of α-synuclein fibrils. A volume of 5 µL of sonicated fibrils was loaded onto a mica slide and incubated for 15 min. The slide was washed three times with 100 µL H_2_O and subsequently dried with N_2_. The sample was measured using a NanoWizard III (JPK BioAFM) with an OMCL-AC160TS cantilever (Olympus) in tapping mode in air. To determine the length distribution, a total number of 547 fibrils were analyzed with ImageJ. The length of each fibril was measured using the ruler tool.

### Immunohistochemical analysis

Formalin fixed brain and spinal cord samples were dehydrated in a series of alcohol and xylene baths, embedded in paraffin, cut into 6-µm-thick coronal sections on an RM2255 microtome (Leica), mounted on glass slides, dried over night, and stored at 4 °C. Tissue sections were first deparaffinized and then rehydrated through incubation in xylene and a series of graded ethanol baths. For antigen retrieval, the slides were incubated in citrate buffer (pH 6.0) for 5 min at room temperature and additionally boiled for 10 min in a microwave oven. After cooling down, endogenous peroxidase activity was inhibited by incubation with a 5% hydrogen peroxide solution in methanol for 30 min at room temperature. Tissue sections were blocked with 20% (v/v) normal goat serum (Thermo Fisher Scientific), 1% (v/v) BSA, and 0.5% Triton X-100 (Sigma) in PBS for 1 h at room temperature. Incubation with the primary antibody was done in 1% (v/v) normal goat serum, 1% (v/v) BSA, and 0.25% Triton X-100 in PBS overnight at room temperature. Antibodies used in this study with corresponding dilutions are listed in Online Resource 1. After washing once with 0.25% (v/v) Triton X-100 in PBS, and twice with PBS, sections were incubated with a secondary, peroxidase-conjugated antibody using the MOM kit (Vector Laboratories) for 1 h at room temperature. The last step was avoided for the biotinylated pSyn#64 antibody against phosphorylated α-synuclein at serine 129 (Wako). After washing once with 0.25% (v/v) Triton X-100 in PBS, and twice with PBS, peroxidase-positive structures were visualized by incubation with DAB (3-3′-diaminobenzidine) for 20–40 s. The reaction was stopped with 3% (v/v) hydrogen peroxide solution. Tissue sections were counterstained with Mayer’s hematoxylin (Merck) and the slides were coverslipped with Vectamount AQ (Vector Laboratories). Visual analysis was performed with ZEN lite software (Carl Zeiss) after scanning the slides with an Axio Scan.Z1 slide scanner (Carl Zeiss).

For quantification of brain areas with staining for phosphorylated α-synuclein, coronal sections at bregma 0.26 mm, − 1.70 mm, − 3.28 mm, and − 6.24 mm were used. Digitalized images were masked prior to analysis. We used a scoring scheme from 0 to 5 [0: no aggregates, 1: sparse (few neurites, no soma), 2: mild (more neurites, max 2 soma), 3: moderate (soma and neurites, but with large areas without aggregates), 4: dense (many neurites and soma), and 5: severe (area covered with pathology)] based on a publication by Rey et al. [42]. For each inoculation route, pathology in each brain region was scored in both brain hemispheres of three animals, and average scores were presented in a colour-coded heat map.

Pathology in the motor cortex was quantified under masked conditions using Fiji V2.0.0 1.49v [46]. Digitalized images of coronal brain tissue sections at bregma 0.26 mm with staining for phosphorylated α-synuclein were analyzed by counting neurites and neuronal somata separately in each layer of the primary and secondary motor cortex for a total of three-to-four animals for each inoculation group.

To assess gliosis, IBA-1- or GFAP-positive staining was each quantified under masked conditions in four separate one square-millimeter-sized areas per coronal section at bregma − 3.28 mm in three-to-four animals per inoculation group. Digitalized images were converted to an 8-bit format and the lower and upper thresholds were set to 0 and 130, respectively. The lower and upper thresholds represent mean values of manually analyzed images for all four inoculation routes from fibril- and BSA-injected animals. The area of IBA-1- or GFAP-positive staining was measured in each image using a Fiji macro. Data are presented as mean percentages ± standard deviation (SD). Groups were compared using one-way ANOVA followed by the Holm-Šídák test using Prism 7 (GraphPad Software). *P* values below 0.05 were considered statistically significant.

### Immunofluorescence analysis

Paraffin-embedded tissues were cut into 6-µm-thick coronal sections, mounted on glass slides, deparaffinized, and rehydrated as indicated before. For antigen retrieval, slides were incubated in citrate buffer (pH 6.0) as previously described, except for the ubiquitin staining, where formic acid treatment was used for antigen retrieval. After cooling down and washing twice with PBS, autofluorescence of the tissue was quenched by incubation in CuSO_4_ for 90 min at room temperature [47]. The slides were blocked in 20% (v/v) normal goat serum, 1% (v/v) BSA, and 0.5% (v/v) Triton X-100 in PBS for 1 h at room temperature. Sections were then incubated with a primary antibody in 1% (v/v) normal goat serum, 1% (v/v) BSA, and 0.25% Triton X-100 in PBS overnight at room temperature. Antibodies used in this study with the corresponding dilutions are listed in Online Resource 1. After washing once with 0.25% (v/v) Triton X-100 in PBS, and twice with PBS, sections were stained with corresponding Alexa Fluor 488- or Alexa Fluor 594-conjugated (Thermo Fisher Scientific) secondary antibodies and the nuclear dye DAPI (4′,6-diamidino-2-phenylindole; Thermo Fisher Scientific) in 1% (v/v) normal goat serum, 1% (v/v) BSA, and PBS for 1 h at room temperature. Slides were coverslipped with Fluoromount medium (Sigma) and visualized with an LSM700 confocal laser-scanning microscope (Carl Zeiss).

### Western blot analysis

Brain and spinal cord samples were homogenized in Ca^2+^- and Mg^2+^-free PBS (pH 7.4) in the presence of protease and phosphatase inhibitors (HALT Protease and Phosphatase Inhibitor Cocktail, Thermo Fisher Scientific) by two 30-s cycles in a Precellys 24-Dual homogenizer (Peqlab) to reach a final concentration of 20% (w/v). Homogenates were adjusted to 750 mM NaCl and centrifuged at 1000×*g* for 5 min at 4 °C to remove debris. Total protein concentration was determined with the Pierce BCA Protein Assay Kit (Thermo Fischer Scientific). For further analysis, 500 µg of total protein was incubated in 10% (w/v) N-lauroylsarcosyl (Sigma) for 15 min on ice. Homogenates were ultracentrifuged at 465,000×*g* for 1 h at 4 °C over a 3 mL 10% (w/v) sucrose cushion in a TLA-110 rotor (Beckman Coulter) [34]. Pellets were resuspended in 50 µL of fresh TD4215 denaturing buffer containing 4% sodium dodecyl sulfate (SDS), 2% β-mercaptoethanol, 192 mM glycine, 25 mM Tris, and 5% (w/v) sucrose. Samples were boiled for 5 min and loaded onto 4–12% NuPage Bis–Tris gels (Thermo Fisher Scientific). SDS–polyacrylamide gel electrophoresis was processed in a morpholineethanesulfonic acid buffer system (Thermo Fisher Scientific). Separated proteins were transferred onto polyvinylidene difluoride membranes using a semidry blotting system and cross-linked in 0.4% (v/v) paraformaldehyde in Tris-buffered saline (Sigma) for 30 min at room temperature. Membranes were blocked in buffer containing TBS with 0.05% (v/v) Tween 20 (MP Biomedical) and 5% (w/v) milk for 1 h at room temperature. Membranes were incubated either with the EP1536Y antibody for phosphorylated α-synuclein (Abcam) or an antibody for GAPDH (Abcam) over night at 4 °C. Following three washing steps with 0.05% (v/v) Tween 20 in TBS, the blots were incubated with an anti-rabbit or anti-mouse horseradish peroxidase-linked secondary antibody (Cayman) at a 1:10,000 dilution for 1 h at room temperature. The chemiluminescent signal was visualized with SuperSignal West Dura Extended Duration Substrate (Thermo Fisher Scientific) in a chemiluminescence reader (Fusion FX, Vilber).

### Quantification of α-synuclein aggregation by time-resolved fluorescence energy transfer (TR-FRET)

The amount of aggregated human α-synuclein in brain and spinal cord samples was measured by TR-FRET using a commercially available kit (Cisbio). Briefly, triplicates of 10-µL samples with 1 µg of total protein were prepared from 20% brain homogenate or 10% spinal cord homogenate in PBS by dilution with lysis buffer 1X. Ten µL of a pre-mixed antibody solution containing the anti-h-α-Synuclein-Tb-Cryptate (donor) and anti-h-α-Synuclein-d2 (acceptor) antibodies were added to each sample. Twenty-µL triplicates of each sample including controls were transferred on a 96-well half-area flat-bottom microplate, covered with a plate sealer, and incubated for 20 h at room temperature. Fluorescence emission was measured at 665 nm for FRET-dependent acceptor fluorescence and at 620 nm for FRET-independent donor fluorescence on a CLARIOstar microplate reader (BMG Labtech). The ratio of both fluorescence emission values multiplied by 10,000 is directly proportional to the amount of human α-synuclein aggregates in each sample.

## Results

### Signs of neurological disease after oral, intravenous, intraperitoneal, or intracerebral challenge of TgM83^+/−^ mice with α-synuclein fibrils

**Fig. 1:**
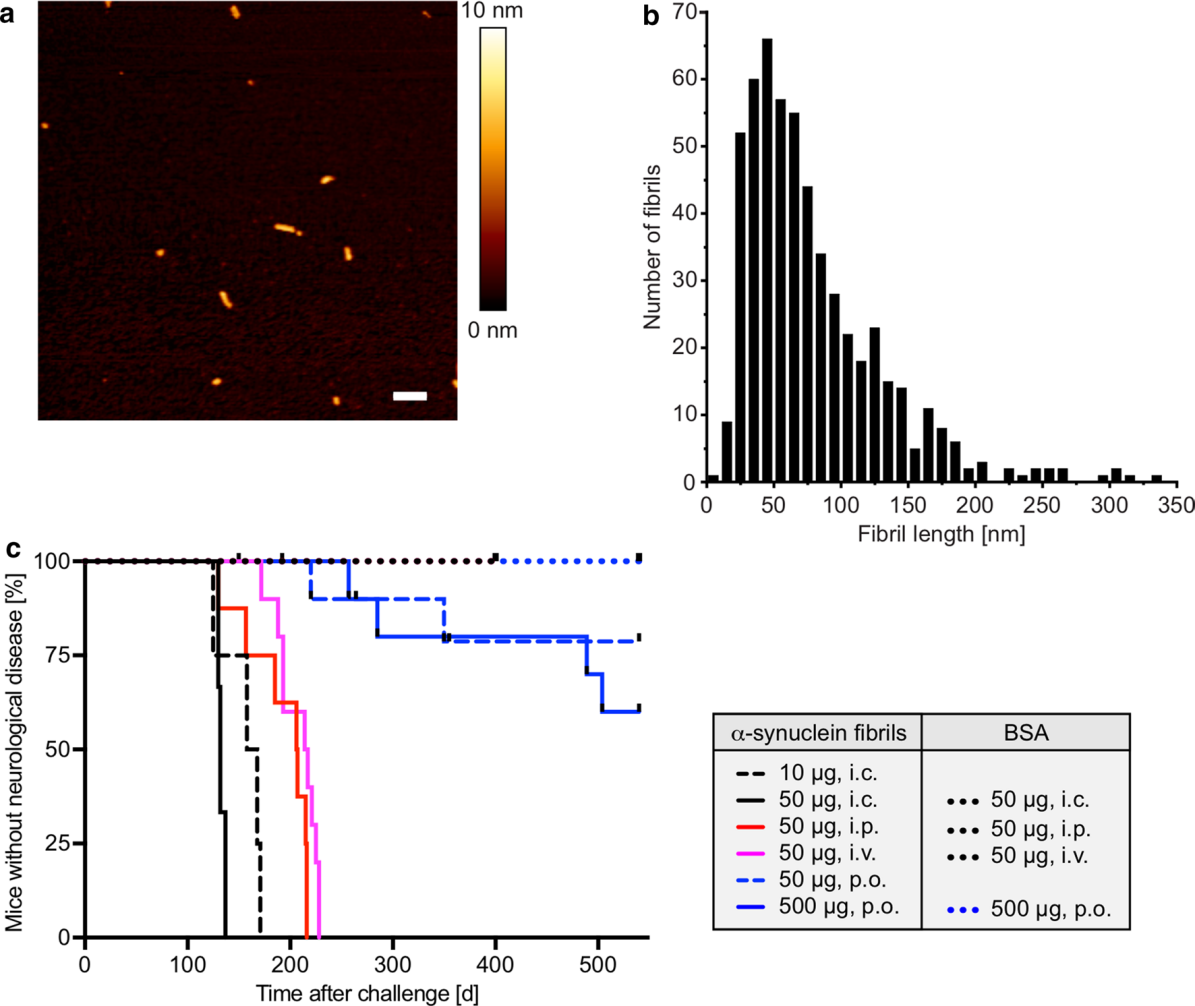
Kaplan–Meier survival curves showing the percentage of healthy animals after oral, intravenous, intraperitoneal, or intracerebral challenge with α-synuclein fibrils or BSA. We used atomic force microscopy to determine the length of the sonicated α-synuclein fibrils with which we challenged TgM83^+/−^ mice. The colour scale indicates the height profile. Scale bar = 200 nm (**a**). Size distribution of the sonicated α-synuclein fibrils as determined by values obtained from statistical analysis of the aggregates identified in the atomic force microscopy images (**b**). Two out of 10 mice developed neurological disease at 220 days and 350 days after oral challenge with 50 µg of α-synuclein fibrils (blue hatched line), and four out of 10 mice within 384 ± 131 days (mean ± SD) after oral challenge with 500 µg (blue solid line). Two additional mice died over night at 264 days and 355 days after inoculation in the low-dose group and two at 150 days and 192 days in the high-dose group without showing signs of neurological disease and without us being able to analyze them for neuropathology. Intravenous challenge of TgM83^+/−^ mice with 50 µg of α-synuclein fibrils caused signs of neurological disease in 10 out of 10 mice in 208 ± 20 days after challenge (magenta line). Comparably, intraperitoneal challenge with 50 µg of α-synuclein fibrils resulted in signs of neurological disease in 10 out of 10 mice in 202 ± 35 days (red line). Intracerebral challenge into the striatum with 10 µg or 50 µg of α-synuclein fibrils was the fastest route to cause signs of neurological disease in 156 ± 20 days (black hatched line) and 133 ± 4 days (black solid line), respectively, in 10 of 10 mice in each group. None of the BSA-challenged control mice developed any sign of neurological disease throughout the course of the experiment (blue dotted lines for oral BSA challenges and black dotted lines for all other BSA challenges) (**c**)

TgM83^+/−^ mice hemizygously express the A53T mutant of human α-synuclein from the *Prnp* promoter and remain free of any spontaneous neuropathology or disease for over 600 days [17]. To test TgM83^+/−^ mice for susceptibility to oral challenge with recombinant, human, wild-type α-synuclein fibrils, we treated groups of 10 6–8-week-old male and female mice once only by oral gavage with a low dose (50 µg) or a high dose (500 µg) of α-synuclein fibrils, or with 500 µg bovine serum albumin (BSA) as a negative control (Fig. 1; Table 1). Whereas none of the BSA-challenged mice developed any sign of neurological disease throughout the course of the 540-day experiment, two out of eight mice in the low-dose group, at 220 days and 350 days, and four out of eight mice in the high-dose group, within 384 ± 131 days (mean ± SD), developed neurological disease with marked signs of paralysis, kyphosis, and reduced activity after oral challenge. Two additional mice died after oral challenge with α-synuclein fibrils in the low-dose group at 264 days and 355 days and two in the high-dose group at 150 days and 192 days without showing signs of neurological disease and without us being able to analyze them for neuropathology. Intravenous challenge of TgM83^+/−^ mice by tail-vein injection with 50 µg of α-synuclein fibrils caused signs of neurological disease in 10 out of 10 mice in 208 ± 20 days after inoculation, whereas challenge of a control group of nine mice with 50 µg BSA via the same route did not result in any sign of neurological disease in over 400 days. Comparably, intraperitoneal challenge by injection with 50 µg of α-synuclein fibrils caused neurological disease in 10 out of 10 mice in 202 ± 35 days. By comparison, intraperitoneal injections of a control group with 50 µg BSA did not result in neurological disease in over 400 days. Intracerebral challenge by stereotactic injection into the striatum with 10 µg, and more so with 50 µg, of α-synuclein fibrils was the fastest route to cause signs of neurological disease in 156 ± 20 days and 133 ± 4 days, respectively, in 10 out of 10 mice in each group. As with the other inoculation routes, intracerebral injection of control mice with 50 µg BSA did not result in signs of neurological disease in over 400 days.

**Table 1:** Incubation times in TgM83^+/−^ mice after challenge with α-synuclein fibrils or BSA

Inoculation route	Inoculum type	Inoculum amount (µg)	Mice with neurological disease/mice inoculated	Mean survival time ± SD (days)
Oral	Fibrils	50	2/8	220 and 350
	Fibrils	500	4/8	384 ± 131
	BSA^a^	500	0/9	≥ 570
Intravenous	Fibrils	50	10/10	208 ± 20
	BSA^a^	50	0/9	≥ 400
Intraperitoneal	Fibrils	50	10/10	202 ± 35
	BSA^a^	50	0/8	≥ 400
Intracerebral	Fibrils	10	10/10	156 ± 20
	Fibrils	50	10/10	133 ± 4
	BSA^a^	50	0/8	≥ 400

^a^*BSA* bovine serum albumin

### Neurologically diseased TgM83^+/−^ mice accumulate species of phosphorylated and aggregated α-synuclein in their CNS

**Fig. 2:**
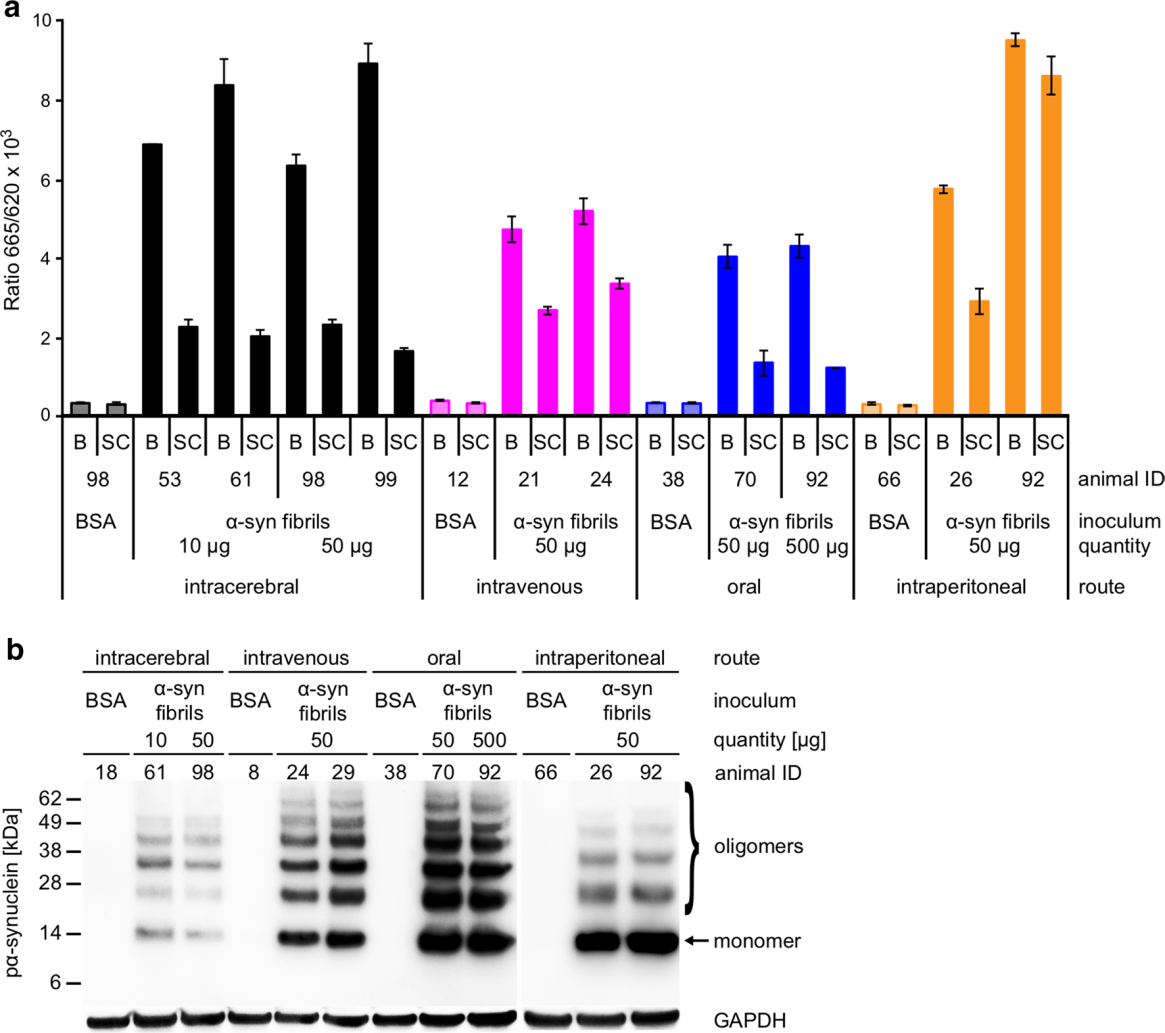
Biochemical analysis of pathological α-synuclein in the CNS of challenged TgM83^+/−^ mice. We quantified the amount of aggregated human α-synuclein in brain and spinal cord homogenates from TgM83^+/−^ mice challenged with α-synuclein fibrils or BSA using a commercially available fluorescence resonance energy transfer (FRET) assay (**a**). In contrast to healthy, BSA-challenged control animals, all animals which had developed neurological symptoms after challenge with α-synuclein fibrils had also accumulated significantly elevated amounts of aggregated α-synuclein in their brain and spinal cord regardless of the route of challenge. Interestingly, the amount of aggregated α-synuclein was frequently higher in the brain than in the spinal cord of each tested animal. Additional biochemical analysis of brain homogenates by western blotting showed that diseased TgM83^+/−^ mice had accumulated sarkosyl-insoluble aggregates of phosphorylated α-synuclein in their brains when probed with the EP1536Y antibody which recognizes α-synuclein that is phosphorylated at Ser129 (**b**). These aggregates presented as several additional high-molecular weight bands above the 15-kDa band of monomeric, phosphorylated α-synuclein. In contrast, the brains of BSA-challenged TgM83^+/−^ control mice were free of sarkosyl-insoluble aggregates of phosphorylated α-synuclein. Molecular weight is shown in kilodalton (kDa). Sample loading in each lane is shown by detection of GAPDH

We quantified the amount of aggregated human α-synuclein in brain and spinal cord homogenates from TgM83^+/−^ mice challenged with α-synuclein fibrils or BSA using a commercially available fluorescence resonance energy transfer (FRET) assay (Fig. 2a). In contrast to healthy, BSA-challenged control animals, all animals with neurological signs of disease tested, including animals after intravenous and oral as well as those after intracerebral and intraperitoneal challenge with α-synuclein fibrils, had accumulated significantly elevated amounts of aggregated α-synuclein in their brain and spinal cord regardless of the route of challenge. Interestingly, the amount of aggregated α-synuclein was frequently higher in the brain than in the spinal cord of each tested animal, although the transgene is expressed at an approximately 5.8-fold higher level in the spinal cord than in the brain [17]. Several conditions could explain this observation. Oligomerization of α-synuclein could be affected by factors that are differentially expressed in brain and in spinal cord resulting in higher brain levels of oligomeric α-synuclein. Alternatively, α-synuclein may start to oligomerize in the spinal cord later than in the brain, because propagating seeds may need longer to reach the spinal cord than the brain. In addition, clearance of oligomeric α-synuclein in the spinal cord could be more efficient than in brain. Additional biochemical analysis of brain homogenates by western blotting showed that diseased TgM83^+/−^ mice had accumulated sarkosyl-insoluble aggregates of phosphorylated α-synuclein in their brains when probed with the EP1536Y antibody which recognizes α-synuclein that is phosphorylated at Ser129 (Fig. 2b). These aggregates presented as several additional high-molecular-weight bands above the 15-kDa band of monomeric, phosphorylated α-synuclein. In contrast, the brains of BSA-challenged TgM83^+/−^ control mice were free of sarkosyl-insoluble aggregates of phosphorylated α-synuclein.

### Diseased TgM83^+/−^ mice display deposits of pathological species of α-synuclein in the brain and spinal cord

**Fig. 3:**
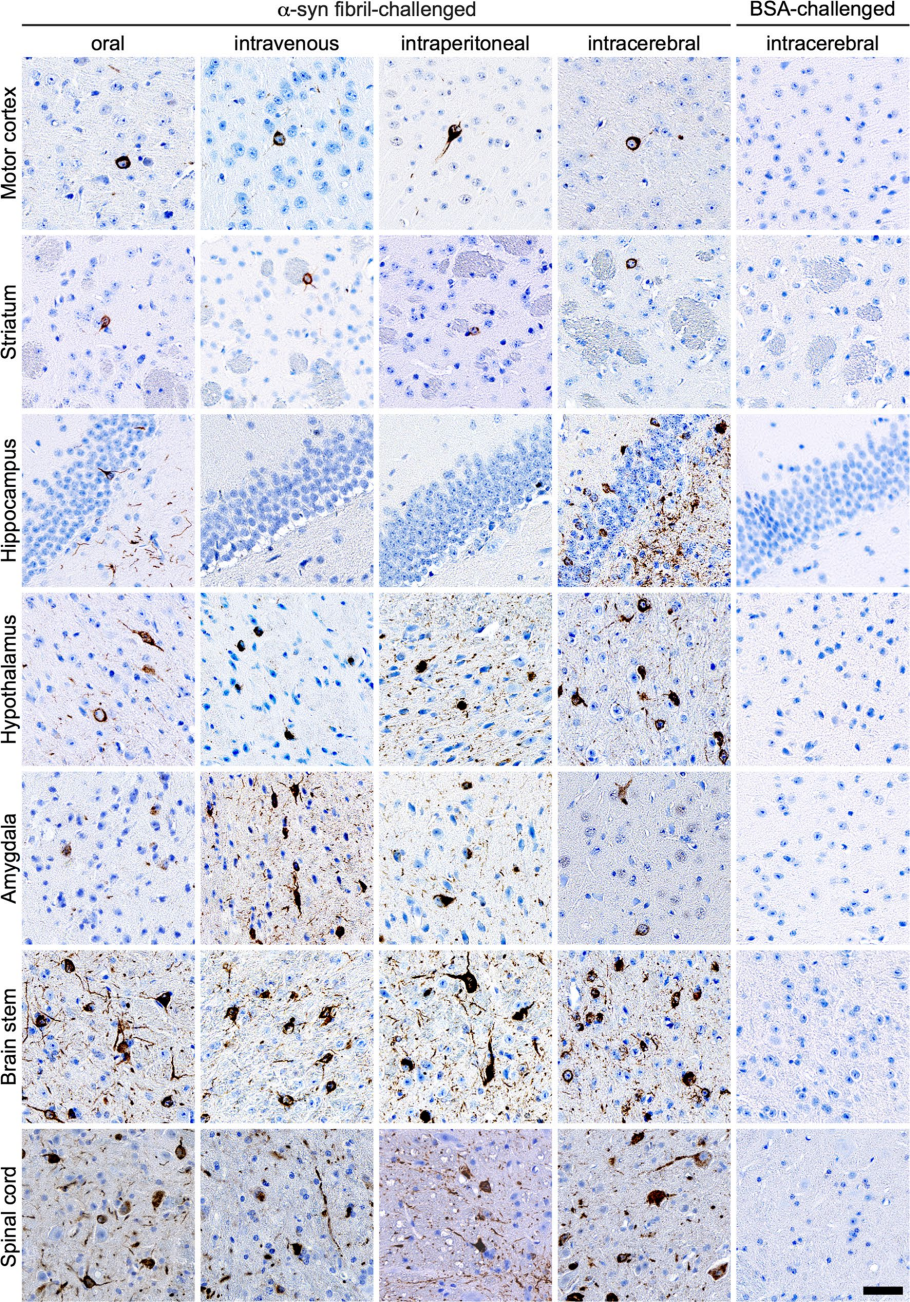
Diseased TgM83^+/−^ mice harbor deposits of pathological α-synuclein in their CNS. To reveal deposits of pathological deposits of α-synuclein in the CNS, we stained brain and spinal cord tissue sections with the pSyn#64 antibody against α-synuclein phosphorylated at Ser129. Diseased animals that had been challenged with α-synuclein fibrils but not control animals challenged with BSA showed abundant deposits of pathological α-synuclein in neuronal cell bodies and neurites in the brain and spinal cord. Deposits of pathological α-synuclein were present throughout the cerebrum including the motor cortex, the striatum, the hypothalamus, the amygdala, and, predominantly, the brain stem. Interestingly, the hippocampus was only affected in animals challenged intracerebrally or orally with α-synuclein fibrils. In the spinal cord, neuronal deposits of phosphorylated α-synuclein were broadly distributed in the gray matter. Scale bar = 50 µm

**Fig. 4:**
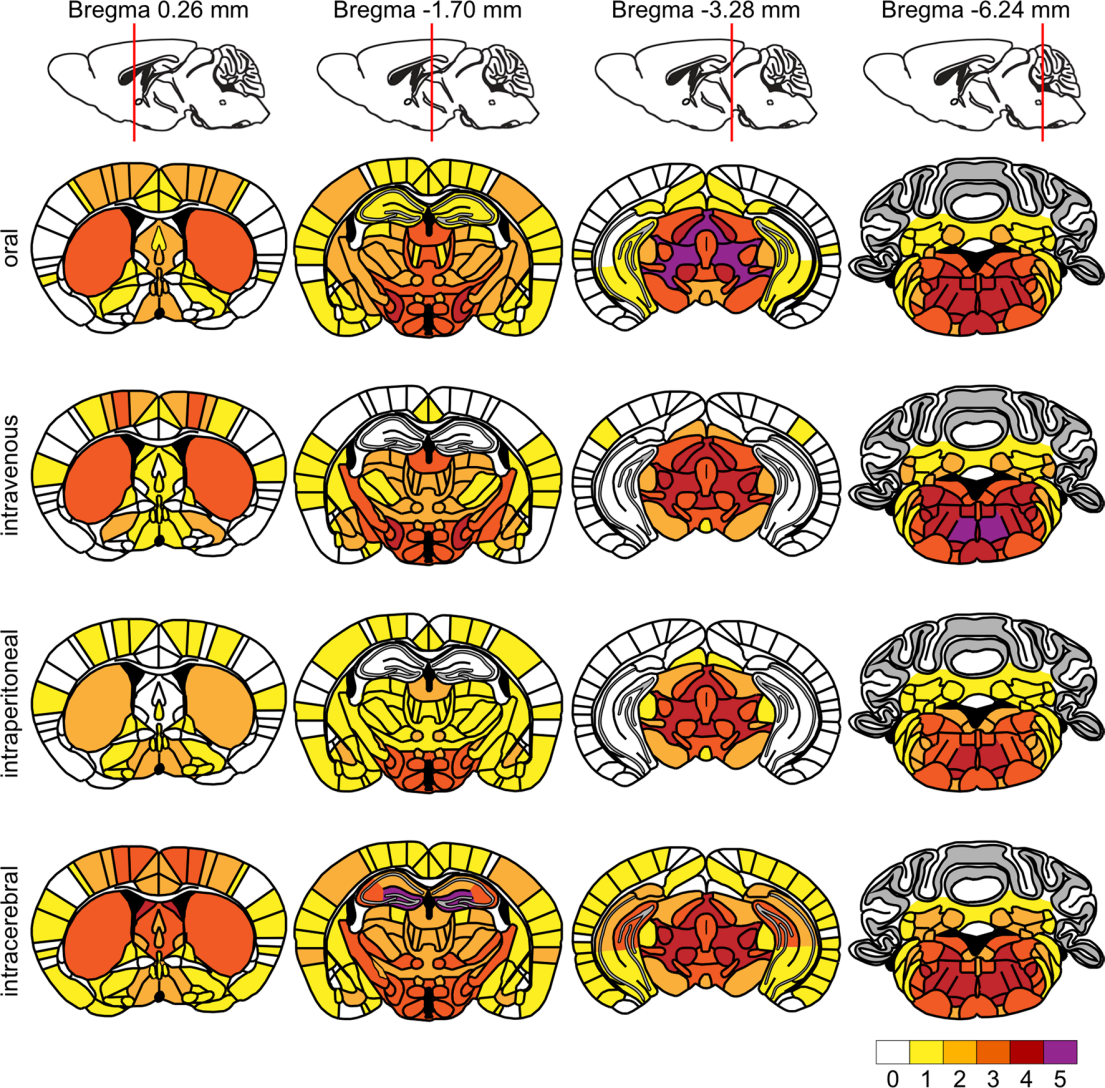
Heat map showing the distribution of phosphorylated α-synuclein in the brains of diseased TgM83^+/−^ mice. We quantitatively analyzed the distribution of phosphorylated α-synuclein in the brains of diseased TgM83^+/−^ mice after oral, intravenous, intraperitoneal, and intracerebral challenge with α-synuclein fibrils and summarized our results on a scale from 0 to 5 reflective of no to severe pathology as a heat map for four coronal brain sections spanning rostral-to-caudal brain areas

Immunohistochemical staining of brain and spinal cord tissue sections with the pSyn#64 antibody against α-synuclein phosphorylated at Ser129 revealed abundant deposits in neuronal cell bodies and neurites in the brain and gray matter of the spinal cord of diseased animals but not in those of control animals challenged with BSA (Fig. 3). To better convey the distribution of pathology in the brain across the four different inoculation groups, we performed a quantitative analysis of the pathology and summarized our findings as a heat map showing the extent of pathology in four different brain regions (Fig. 4). Deposits of pathological α-synuclein were present throughout all four analyzed brain regions and generally increased in a rostral-to-caudal pattern for every route of challenge. At bregma 0.26 mm, most cortical regions did not show any or just sparse deposits of phosphorylated α-synuclein, except for the primary and secondary motor cortex, where more neurites and somata were affected regardless of the route of inoculation (Online Resource 2). We detected phosphorylated α-synuclein also in the striatum and the lateral septal nucleus of all four groups, but intraperitoneally challenged animals showed less pathology than others. At bregma − 1.70 mm, affected neurites were present throughout most of the cortical regions and displayed less pathology in intravenously injected animals. Between the four groups, a comparable amount of α-synuclein pathology was observed in interbrain regions with sparse-to-mild pathology in the amygdala, mild pathology throughout the thalamus, and moderate pathology throughout the hypothalamus. For orally and intravenously challenged mice, we observed dense pathology in the peduncular part of the lateral hypothalamus. The hippocampus was not affected after intravenous or intraperitoneal challenge. However, after oral challenge, we observed sparse pathology, and after intracerebral challenge mild pathology in the CA1 region, moderate pathology in the CA3 region, and severe pathology in the dentate gyrus. At bregma − 3.28 mm, cortical regions were devoid of pathology, except for brains of intracerebrally challenged mice, where sparse pathology was present in almost all cortical regions. In orally challenged mice the distribution of α-synuclein pathology within the hippocampus was sparse, and in intracerebrally challenged mice sparse-to-mild in the CA1 and CA3 regions, and moderate in the dentate gyrus. All four inoculation groups showed an increased amount of pathology in the midbrain with sparse-to-mild pathology in the medial geniculate nuclei, mild-to-moderate pathology in the parabrachial pigmented nucleus of the ventral tegmental area, and mild pathology in the substantia nigra, which was moderate after intravenous challenge. The superior colliculus showed the same distribution pattern of pathology for all routes of challenge, increasing from mild-to-dense for intraperitoneally and intracerebrally challenged animals, from moderate-to-dense for intravenously challenged animals, and from moderate-to-severe for orally challenged animals. For all routes of challenge, the inner midbrain regions showed moderate-to-dense pathology in the lateral periaqueductal gray, the anterior pretectal nucleus, the parvicellular part of the red nucleus, and the mesencephalic reticular formation, except for orally challenged animals, where we observed severe pathology in the mesencephalic reticular formation. At bregma − 6.24 mm, we observed sparse-to-mild pathology in the cerebellar nuclei of all groups but not in the cerebellum. Sparse pathology was also detected in the outer regions of the hindbrain, such as the inferior cerebellar peduncle and the spinal trigeminal tract. In contrast, moderate-to-dense pathology was present in the facial and the raphe magnus nuclei of all groups. The reticular nucleus presented with dense pathology in almost all groups, except for intravenously challenged animals, where the pathology was severe. In general, the distribution of pathology did not differ greatly between animals that were challenged via different routes. One exception was the hippocampus, especially the dentate gyrus, showing pathology only after intracerebral and oral challenge but not after intravenous or intraperitoneal challenge with α-synuclein fibrils. Overall, intraperitoneal challenge resulted in less pathology.

**Fig. 5:**
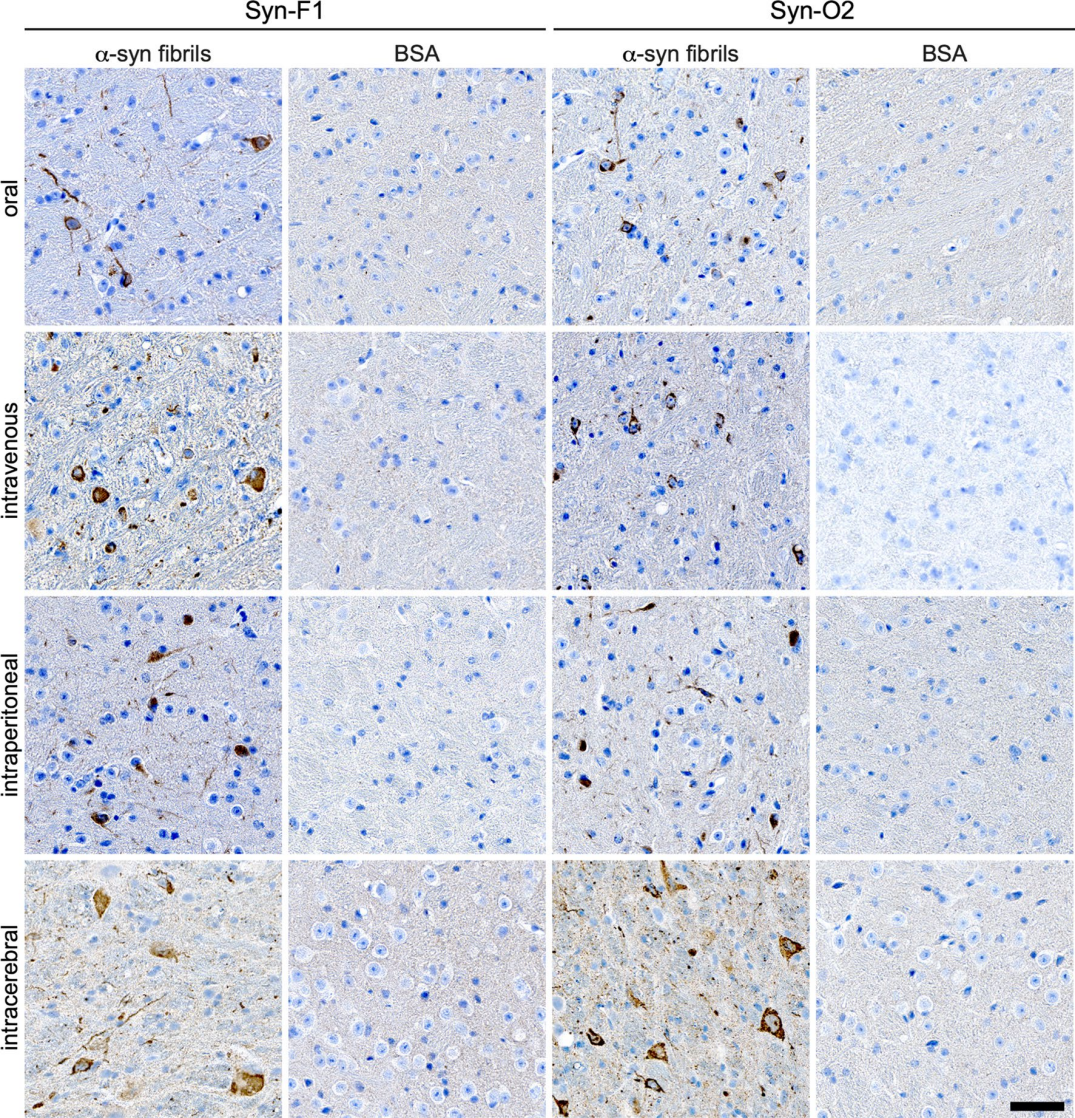
Deposits of pathological α-synuclein in the CNS of diseased TgM83^+/−^ mice have a fibrillar component. Immunohistochemical staining of tissue sections of the brain stem with the Syn-F1 and Syn-O2 antibodies that specifically recognize fibrillar α-synuclein species clearly show that these were part of the pathological α-synuclein deposits located in the soma and neurites of affected neurons in the brain stem. This was observed regardless of the route of challenge. None of the BSA-challenged animals accumulated deposits of fibrillar α-synuclein in the CNS. Scale bar = 50 µm

Deposits of pathological α-synuclein in the CNS of diseased animals did not only consist of phosphorylated α-synuclein, but represented mature amyloid fibrils as detected with the Syn-F1 and Syn-O2 antibodies (Fig. 5), which specifically bind to fibrillar species of α-synuclein [58]. Both antibodies, Syn-F1 and Syn-O2, did not reveal any deposits of pathological α-synuclein in the CNS of BSA-challenged control mice.

### Aggregates of pathological α-synuclein colocalize with ubiquitin and p62 in the CNS

**Fig. 6:**
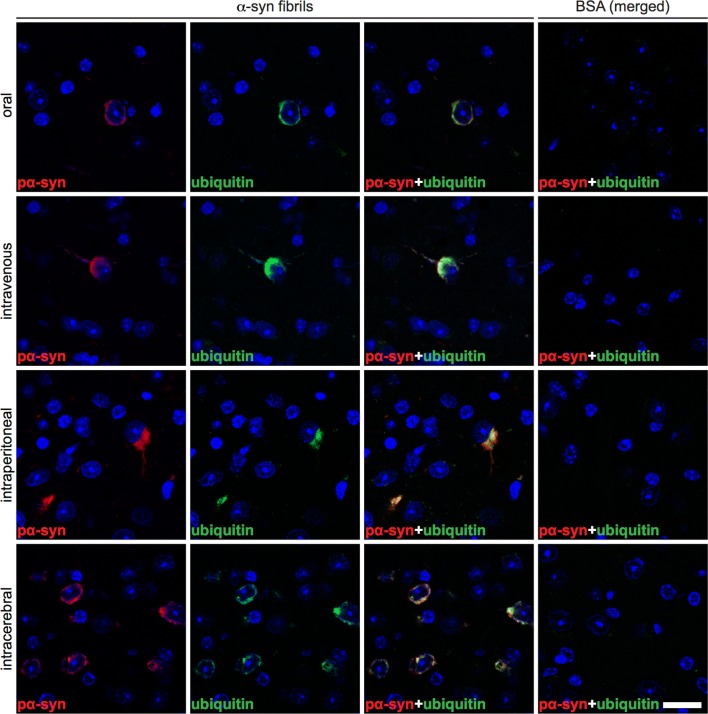
Deposits of phosphorylated α-synuclein colocalize with ubiquitin in the brain of diseased TgM83^+/−^ mice. Immunofluorescence staining of tissue sections of the brain stem show that phosphorylated α-synuclein (red), detected with the EP1536Y antibody, and ubiquitin (green) colocalize in affected neurons of diseased mice. We observed similar results regardless of the route of challenge. Neurons of BSA-challenged mice, for which only merged images are shown, did not accumulate any excessively phosphorylated α-synuclein or ubiquitinated protein deposits. Nuclear staining with DAPI is shown in blue. Scale bar = 20 µm

**Fig. 7:**
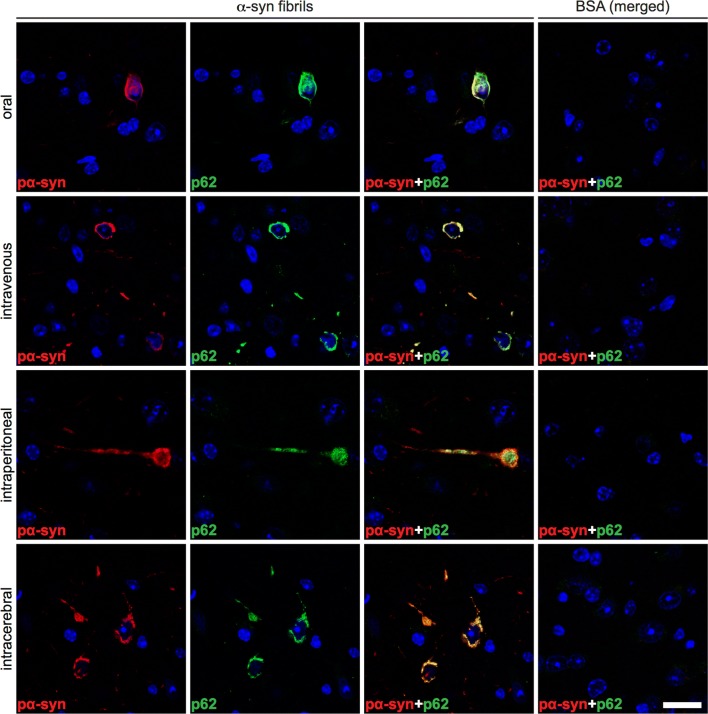
Deposits of phosphorylated α-synuclein colocalize with p62 in the brain of diseased TgM83^+/−^ mice. Immunofluorescence staining of tissue sections of the brain stem show that phosphorylated α-synuclein (red), detected with the pSyn#64 antibody, and p62 (green) colocalize in affected neurons of diseased mice. We observed similar results regardless of the route of challenge. Neurons of BSA-challenged mice, for which only merged images are shown, did not accumulate any excessively phosphorylated α-synuclein or p62-tagged protein deposits. Nuclear staining with DAPI is shown in blue. Scale bar = 20 µm

Studies in patients with PD and MSA and in animal models of these diseases have shown that deposits of pathological α-synuclein in Lewy bodies and glial cytoplasmic inclusions specifically colocalize with markers of the cellular protein degradation machinery, e.g., ubiquitin and p62, which have failed to efficiently target pathological α-synuclein for degradation [28, 30, 33]. Indicative of an abnormal protein homeostasis of α-synuclein, immunofluorescence staining of brain and spinal cord tissue sections for phosphorylated α-synuclein and ubiquitin revealed that these two proteins colocalized in the CNS of diseased animals but not of BSA-challenged control animals (Fig. 6 and Online Resource 3). Concordantly, immunofluorescence staining of brain and spinal cord tissue sections showed that phosphorylated α-synuclein also colocalized with p62 in the CNS of diseased TgM83^+/−^ mice but not of BSA-challenged, healthy control animals (Fig. 7 and Online Resource 4).

### Deposition of pathological α-synuclein triggers neuroinflammation of the CNS

**Fig. 8:**
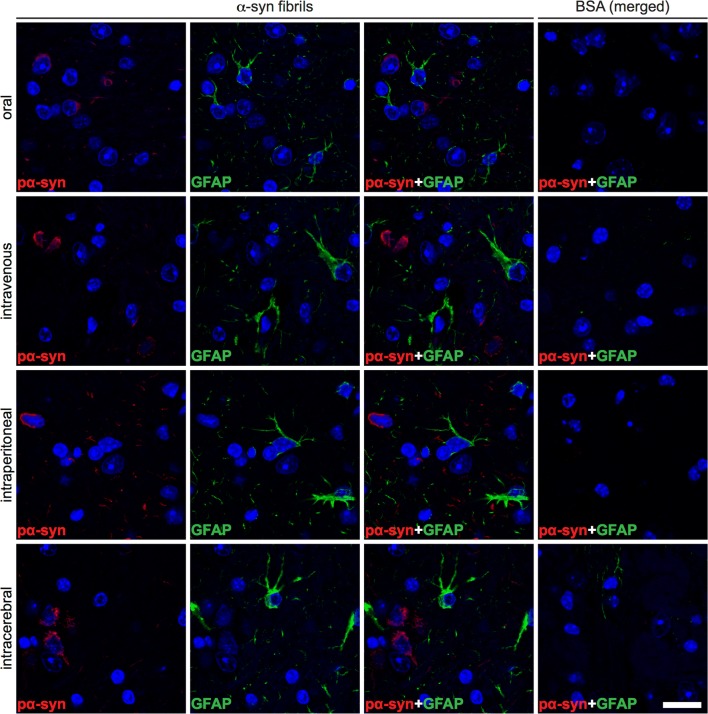
Astrogliosis in the brains of diseased TgM83^+/−^ mice. Immunofluorescence staining of tissue sections of the brain stem with an antibody against GFAP, a marker for astrocytes, shows that neurons of diseased TgM83^+/−^ mice with deposits of phosphorylated α-synuclein (red), which were detected with the pSyn#64 antibody, were frequently surrounded by reactive astrocytes (green). In contrast, no excessive abundance of phosphorylated α-synuclein or reactive astrocytes was observed in the brains of BSA-challenged, healthy control mice. Nuclear staining with DAPI is shown in blue. Scale bar = 20 µm

**Fig. 9:**
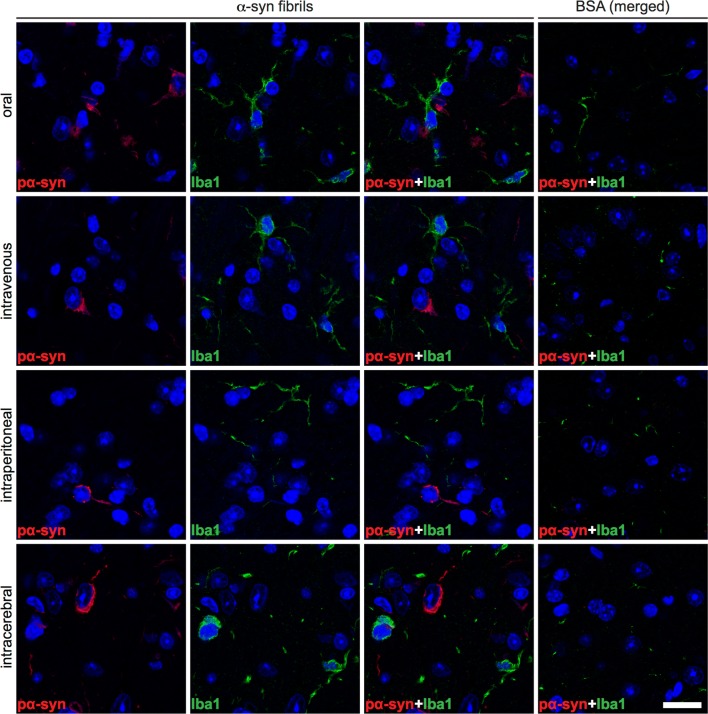
Microgliosis in the brains of diseased TgM83^+/−^ mice. Immunofluorescence staining of tissue sections of the brain stem with an antibody against IBA-1, a marker for microglia, shows that neurons of diseased TgM83^+/−^ mice with deposits of phosphorylated α-synuclein (red), which were detected with the pSyn#64 antibody, were frequently surrounded by activated microglia (green) with an amoeboid morphology. In contrast, no excessive abundance of phosphorylated α-synuclein or activated microglia was observed in the brains of BSA-challenged, healthy control mice. Nuclear staining with DAPI is shown in blue. Scale bar = 20 µm

**Fig. 10:**
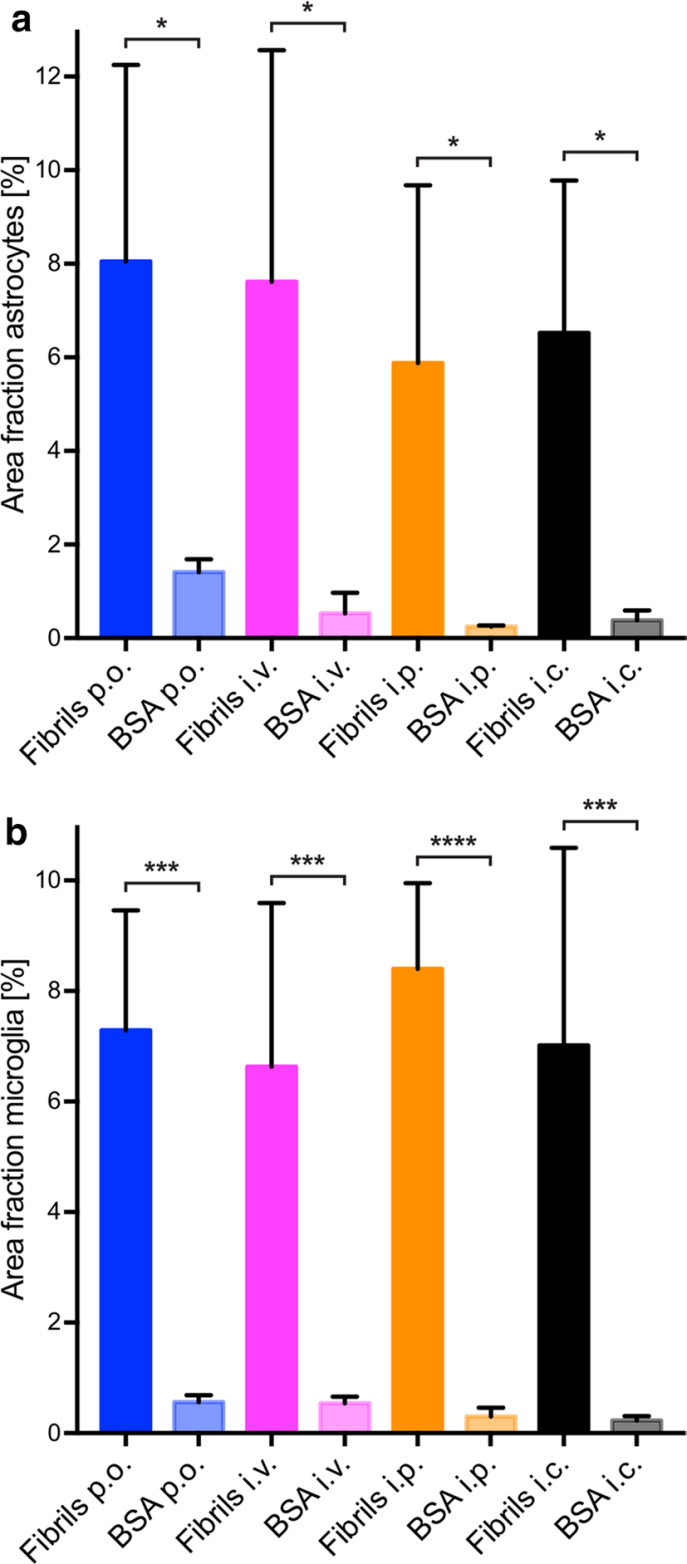
Quantification of gliosis in the brains of challenged TgM83^+/−^ mice. Quantification of brain tissue sections positive for GFAP (**a**) or IBA-1 (**b**) after immunostaining reveal that astrogliosis and microgliosis were significantly induced in animals challenged with α-synuclein fibrils in comparison with BSA-challenged animals regardless of the inoculation route. Data are presented as mean percentage ± standard deviation and are derived from three to four animals per inoculation group. Groups were compared using one-way ANOVA followed by the Holm-Šídák test. *P* values below 0.05 were considered statistically significant (* ≤ 0.05, *** ≤ 0.001, **** ≤ 0.0001)

To detect neuroinflammatory changes in the CNS of diseased TgM83^+/−^ mice that may be attributable to deposits of pathological α-synuclein, we stained brain and spinal cord tissue sections for phosphorylated α-synuclein and for the astrocyte marker glial fibrillary acidic protein (GFAP). The CNS of diseased animals showed marked astrogliosis in the vicinity of neurons with deposits of phosphorylated α-synuclein (Fig. 8 and Online Resource 5). In contrast, the CNS of healthy, BSA-challenged control mice was free of pathological α-synuclein and, equally, devoid of reactive astrocytes. Immunofluorescence staining of brain and spinal cord tissue sections for phosphorylated α-synuclein and IBA-1, which is a marker for microglia, revealed that neurons with deposits of pathological α-synuclein were frequently surrounded by amoeboid microglia characteristic of microgliosis in the CNS of diseased animals (Fig. 9 and Online Resource 6). The CNS of healthy, BSA-challenged control animals was free of any α-synuclein-related pathology and only showed ramified microglia. CNS gliosis was significantly induced regardless of the route of inoculation with α-synuclein fibrils and did not notably differ in intensity between the different inoculation routes (Fig. 10). Overall these findings suggest that neurological disease in TgM83^+/−^ mice that have been challenged with α-synuclein fibrils is characterized by the phosphorylation and aggregation of α-synuclein in neurons, which results in neuroinflammation of the CNS. Importantly, α-synuclein fibrils do not only induce neurological disease in TgM83^+/−^ mice after intracerebral or intraperitoneal injection but also after oral or intravenous transmission.

## Discussion

Our data demonstrate that a single oral or intravenous challenge with α-synuclein fibrils can induce neurological disease in TgM83^+/−^ mice, as we have previously shown for intraglossal and intraperitoneal challenge [10]. The ensuing synucleinopathy was defined by deposits of sarkosyl-insoluble, hyperphosphorylated, and fibrillar α-synuclein species that accumulated in the soma and in neurites of affected neurons in the CNS, reminiscent of Lewy bodies and Lewy neurites that are characteristic for PD [52]. Moreover, aggregated α-synuclein in these pathological deposits colocalized with ubiquitin and p62, as seen in the brains of PD patients, indicating that tagging of pathological α-synuclein for cellular degradation was not successful and resulted in aberrant protein homeostasis [28, 30]. Pathological accumulation of non-degradable and toxic α-synuclein species led to neuroinflammation of the CNS, as evidenced by reactive astrogliosis and microgliosis in the vicinity of neurons with pathological deposits, and, eventually, to neurological disease with signs of motor dysfunction, such as ataxia and paralysis.

Among the four different routes of challenge that we assessed here, intracerebral challenge with α-synuclein fibrils was the fastest route to cause disease with full penetrance. In comparison with intracerebral challenge, intraperitoneal and intravenous challenges with α-synuclein fibrils were slower in causing disease. Although pathology was generally weaker after intraperitoneal challenge, comparable incubation times and full penetrance suggest that neuroinvasion after intraperitoneal and intravenous challenges may occur along the same routes. Oral challenge with α-synuclein fibrils was the slowest transmission route and did not reach full penetrance. Considering that most of the orally administered α-synuclein fibrils were probably digested or shed in feces after passing through the digestive tract, it is remarkable that a sufficient amount of α-synuclein fibrils was able to cause CNS disease in up to 50% of the challenged animals. Intragastric digestion followed by dilution and shedding of α-synuclein fibrils in feces likely lowered the infective dose in comparison with transmission via the other routes resulting in a lower transmission rate after oral challenge. End-point titrations of prions in animals show that transmission rates diminish when the inoculum is diluted [15, 41]. The underlying mechanism of how exactly pathological α-synuclein propagates to the CNS after peripheral challenge has yet to be solved. Staging of PD has shown that the brain stem, next to the olfactory bulb, is one of the two sites, where deposits of pathological α-synuclein are first detected in the CNS and from where pathology further spreads to connected CNS regions [8]. Additional observations in patients with Lewy pathology in neurons of the plexus submucosus and plexus myentericus of the gastrointestinal tract imply that in some patients PD may begin in the “gut” and that pathological α-synuclein reaches the dorsal motor nucleus in the brain stem by retrograde transsynaptic transport along the vagus nerve, which connects the brain stem to the enteric plexuses [18, 19]. In addition, a Danish epidemiological study reported a decreased risk of PD in patients after bilateral vagotomy when compared to the general population (overall adjusted hazard ratio = 0.85; 95% confidence interval: 0.63–1.14; follow-up > 20 years, adjusted hazard ratio = 50.53; 95% confidence interval: 0.28–0.99), which suggests that the vagal nerve may be critically involved in the pathogenesis of PD [54]. Experimental studies in rats that were injected with pathological α-synuclein into the myenteric plexus showed that pathological α-synuclein can indeed be transported via the vagus nerve to the brain [23]. Our data suggest that simple oral ingestion of α-synuclein fibrils is sufficient for them to cross the epithelial lining of the gastrointestinal tract before they are taken up by neurons within the enteric plexuses and transported via the vagus nerve to the brain [9, 61]. Transport by blood followed by crossing of the blood–brain barrier is probably the fastest route by which pathological α-synuclein reaches the CNS after intravenous challenge. This is supported by findings in rats which accumulated fluorescently labeled α-synuclein fibrils in the CNS after repeated intravenous injections of the labeled fibrils for every 2 weeks over a period of 4 months [36]. In contrast to our findings, intravenous challenge of 2-month-old homozygous TgM83^+/+^ mice with only 20 µg of mouse α-synuclein fibrils by another group did not result in signs of neurological disease within 120 days, which may be due to the shorter period of observation, the lower fibril dose, or other properties of the fibrils, such as their size, conformation, or species origin [2]. Nevertheless, histologically, a synucleinopathy was observed in the CNS of all five challenged mice at 120 days after challenge, which was absent from the brains of uninjected, age-matched control mice.

The infectious properties of α-synuclein fibrils that we have characterized here are, as far as possible transmission routes and efficiencies are concerned, comparable to those of PrP prions. Known mechanisms of peripheral neuroinvasion of the CNS by PrP prions include retrograde transport of infectivity along peripheral nerves, especially, along the vagus nerve to the brain, or along the splanchnic nerves to the spinal cord and then to the brain. These routes can be engaged after intraperitoneal transmission, or after oral transmission and passage of prions to the enteric nervous system after crossing the mucosal barrier of the gastrointestinal tract [4, 6, 22]. Alternatively, after oral or intravenous transmission, prions can be transported to the CNS also by blood, where they seem to be able to penetrate the blood–brain barrier [49, 56].

In conclusion, our results demonstrate that α-synuclein can adopt a pathological conformation with infectious and neuroinvasive properties that leads to neuropathology and CNS disease after oral, intravenous, intraperitoneal, or intracerebral delivery which in our opinion qualifies α-synuclein as a prion-like protein. Unlike for human PrP prions that cause kuru, CJD, or variant CJD, natural or iatrogenic transmission of pathological α-synuclein to or between humans has not been reported to date [16, 38, 55, 57]. Therefore, possible incubation periods after human infection with pathological α-synuclein are not known and can only be estimated. Considering that the prodromal phase of PD during which Lewy pathology can be detected in peripheral organs of PD patients can span 10–20 years, it is conceivable that maximal incubation periods after peripheral infection with pathological α-synuclein could take several decades to result in PD [53]. Such prolonged incubation periods after peripheral infection with PrP prions are not unheard of and can take up to 40 or 50 years in some cases, for example, with CJD or kuru prions [14, 43]. There is not a single epidemiological study that assesses the subsequent risk of PD after peripheral transmission of pathological α-synuclein by blood transfusion, for instance, for such long incubation periods. In addition, because PD is the second most common neurodegenerative disease, the prevalence makes it more difficult to observe possible cases of transmission. While PrP prion diseases are quite rare, and extensive surveillance is conducted to look for cases of horizontal transmission, similar changes in disease frequency would be much more complicated to observe in PD diagnoses. Consequently, as a precautionary measure to reduce human exposure to an infectious agent, we recommend heightened biosafety levels for the safe laboratory handling of samples containing pathological forms of α–synuclein.

## Electronic supplementary material

Below is the link to the electronic supplementary material.

Supplementary material 1 (PDF 56 kb)

Supplementary material 2 (TIFF 19701 kb)

Supplementary material 3 (TIFF 15392 kb)

Supplementary material 4 (TIFF 15392 kb)

Supplementary material 5 (TIFF 15392 kb)

Supplementary material 6 (TIFF 15392 kb)

Supplementary material 7 (PDF 80 kb)
