# Supplementary material for: Oral and intravenous transmission of α-synuclein fibrils to mice
Source: Acta Neuropathol. 2019 Jun 22;138(4):515–33. doi: 10.1007/s00401-019-02037-5 (PMC6778172; doi:10.1007/s00401-019-02037-5)
Supplement: Supplementary file 1 — Supplementary material 1 (PDF 56 kb) [file 401_2019_2037_MOESM1_ESM.pdf]

**Table S1** Antibodies used for immunofluorescence, immunohistochemistry, and western blotting

| Target (alternative name) [antibody clone] | Source | Catalogue number | Host | Dilution (IF/IHC) | Antigen retrieval | Dilution (WB) |
|---|---|---|---|---|---|---|
| alpha-Synuclein (fibrillar) [Syn-F1] | Biolegend | 847802 | Mouse | 1:500 | Citrate buffer | – |
| alpha-Synuclein (oligomeric and fibrillar) [Syn-O2] | Biolegend | 847602 | Mouse | 1:500 | Citrate buffer | – |
| alpha-Synuclein (phospho S129) [EP1536Y] | Abcam | AB51253 | Rabbit | 1:200 | Citrate buffer | 1:1000 |
| alpha-Synuclein (phospho S129) [pSyn#64] | Wako | 015-25191 | Mouse | 1:1200 | Citrate buffer | – |
| alpha-Synuclein (phospho S129) [pSyn#64], biotin-conjugated | Wako | 010-26481 | Mouse | 1:1000 | Citrate buffer | – |
| GAPDH | Abcam | ab226408 | | | | 1:1000 |
| Glial fibrillary acidic protein (GFAP) | Dako | Z0334 | Rabbit | 1:400 | Citrate buffer | – |
| Iba1 | Wako | 019-19741 | Rabbit | 1:500 | Citrate buffer | – |
| p62 (sequestosome-1) | Proteintech | 18420-1-AP | Rabbit | 1:100 | Citrate buffer | – |
| Ubiquitin (Ubi-1) | Merck Millipore | MAB1510 | Mouse | 1:500 | Formic acid | – |
