# Supplementary figures and images for: Oral and intravenous transmission of α-synuclein fibrils to mice
Source: Acta Neuropathol. 2019 Jun 22;138(4):515–33. doi: 10.1007/s00401-019-02037-5 (PMC6778172; doi:10.1007/s00401-019-02037-5)

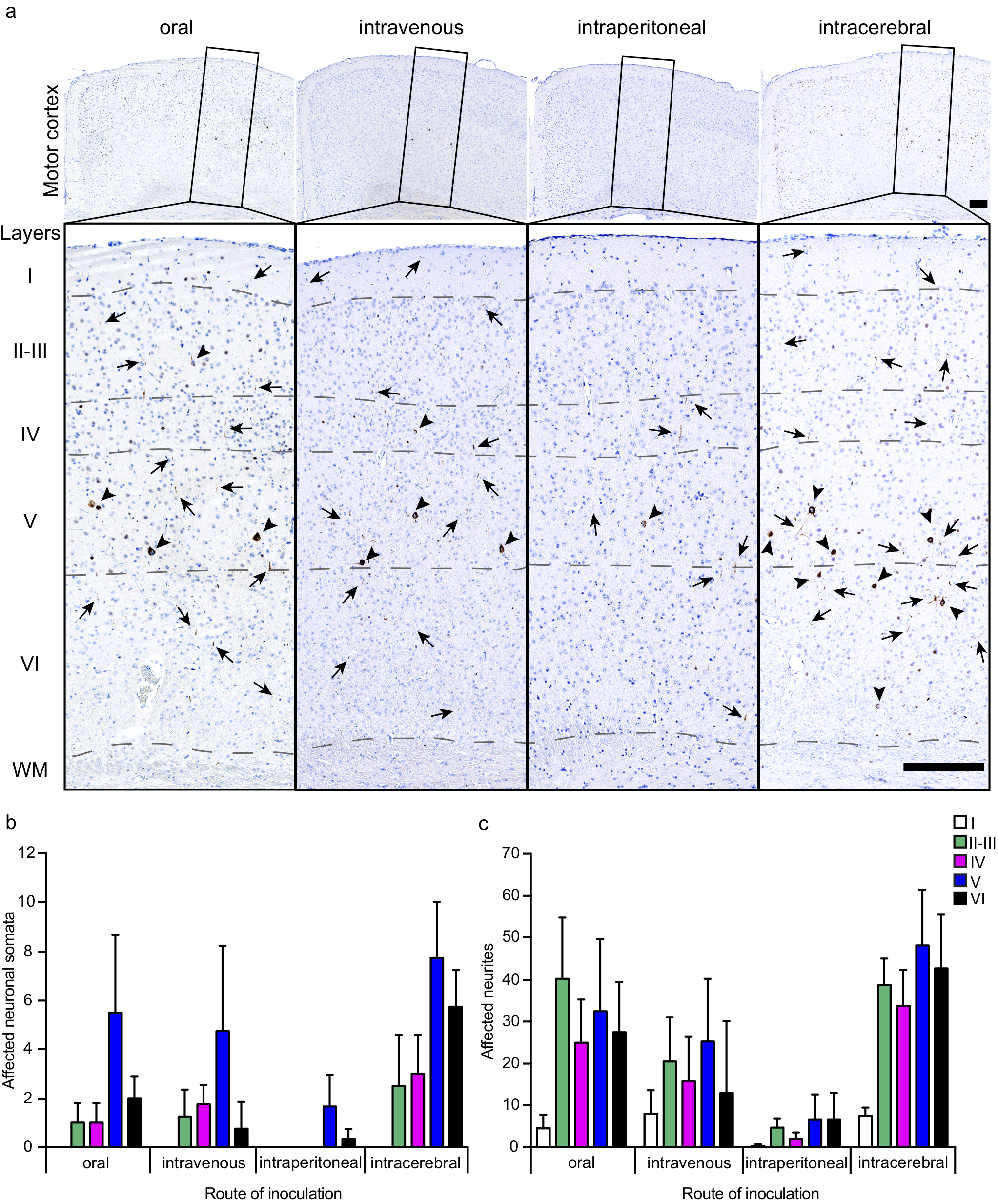

Supplement: Supplementary file 2 — Supplementary material 2 (TIFF 19701 kb) [file 401_2019_2037_MOESM2_ESM.tiff]

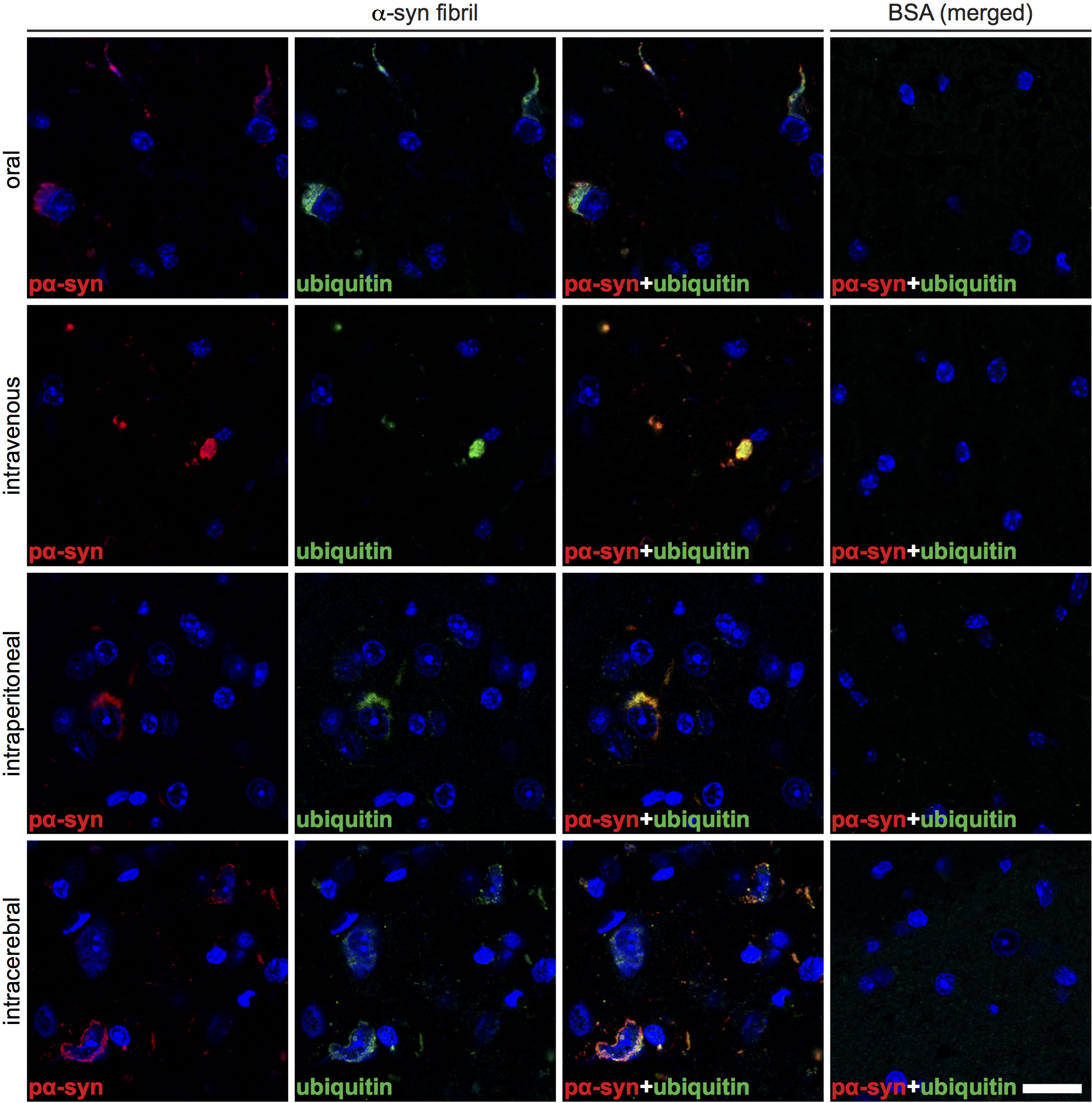

Supplement: Supplementary file 3 — Supplementary material 3 (TIFF 15392 kb) [file 401_2019_2037_MOESM3_ESM.tiff]

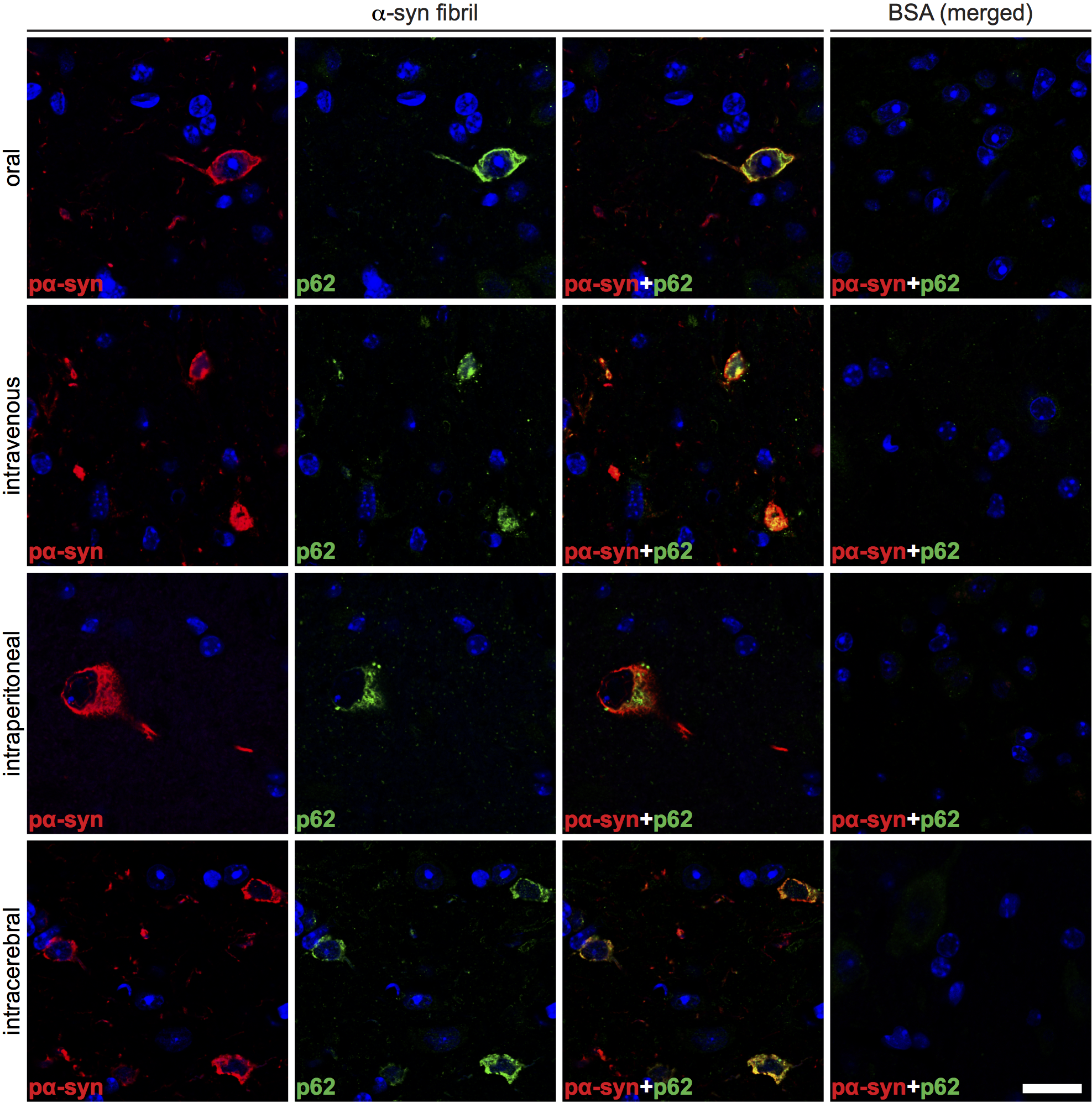

Supplement: Supplementary file 4 — Supplementary material 4 (TIFF 15392 kb) [file 401_2019_2037_MOESM4_ESM.tiff]

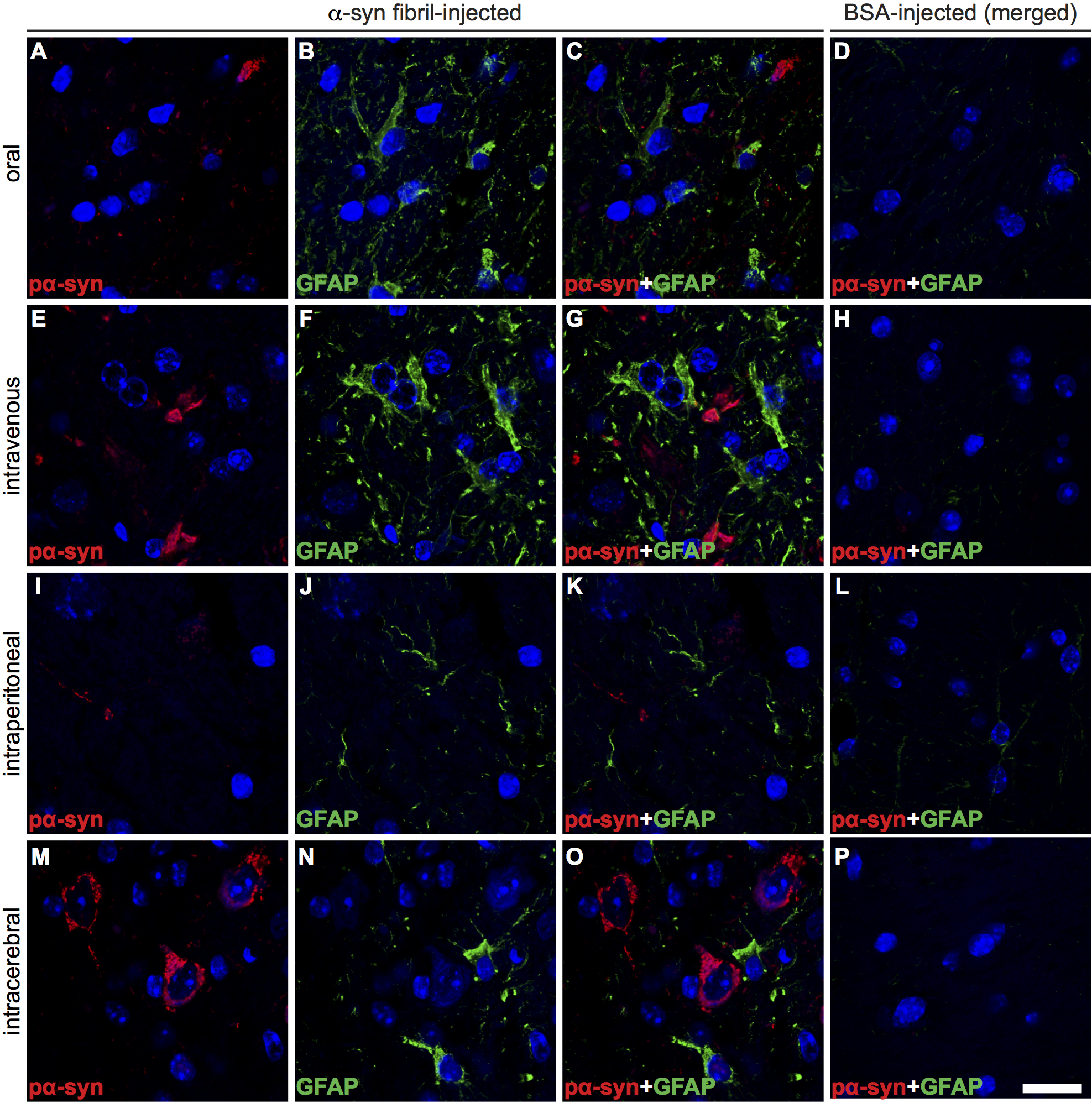

Supplement: Supplementary file 5 — Supplementary material 5 (TIFF 15392 kb) [file 401_2019_2037_MOESM5_ESM.tiff]

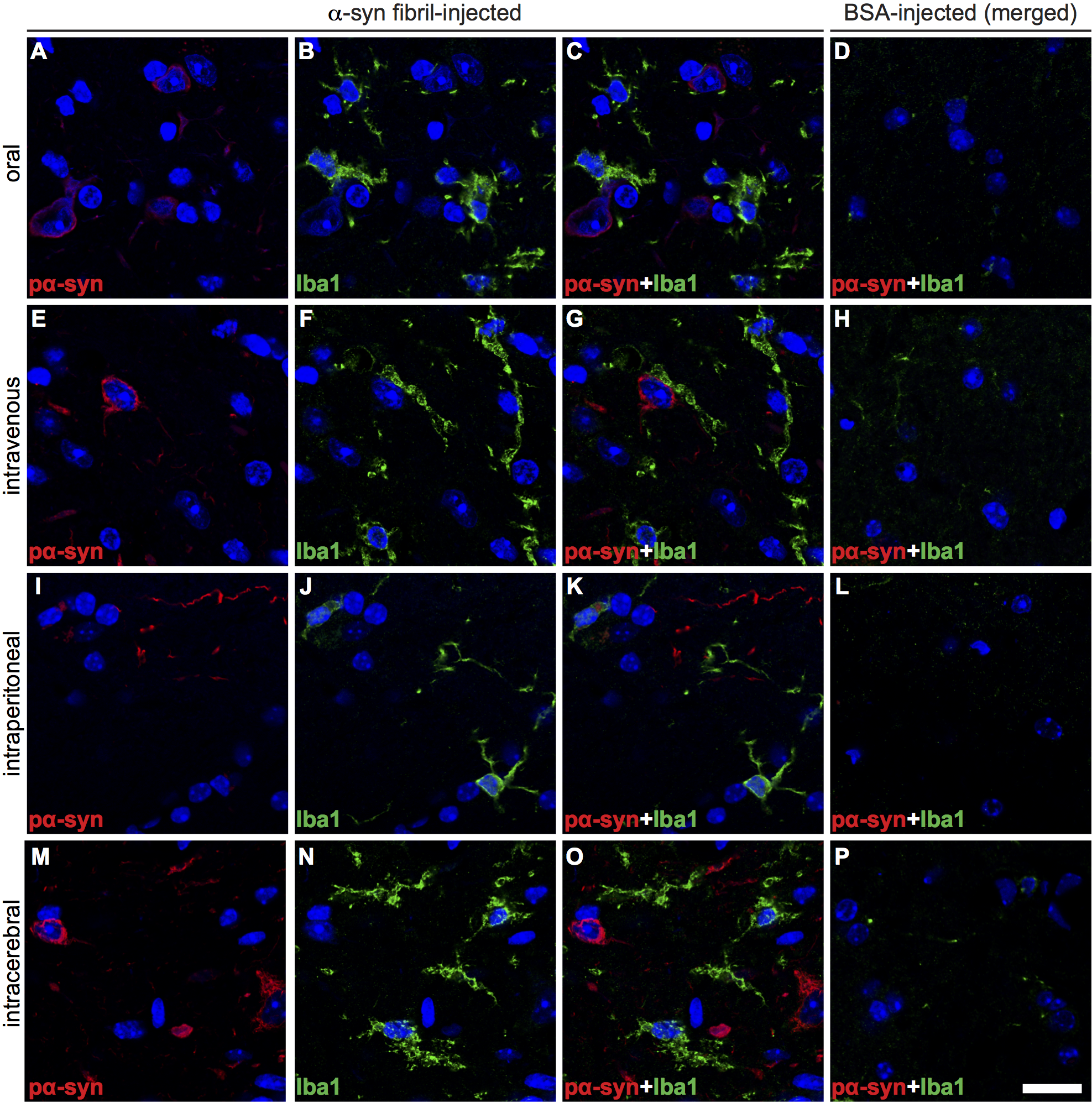

Supplement: Supplementary file 6 — Supplementary material 6 (TIFF 15392 kb) [file 401_2019_2037_MOESM6_ESM.tiff]
