# Supplementary material for: Oral and intravenous transmission of α-synuclein fibrils to mice
Source: Acta Neuropathol. 2019 Jun 22;138(4):515–33. doi: 10.1007/s00401-019-02037-5 (PMC6778172; doi:10.1007/s00401-019-02037-5)
Supplement: Supplementary file 7 — Supplementary material 7 (PDF 80 kb) [file 401_2019_2037_MOESM7_ESM.pdf]

Oral and intravenous transmission of α-synuclein fibrils to mice

Acta Neuropathologica

Stephanie Lohmann,[1] Maria E. Bernis,[1] Babila J. Tachu,[1] Alexandra Ziemski,[3] Jessica Grigoletto,[1] and Gültekin Tamgüney[1,2,3]

1 German Center for Neurodegenerative Diseases (DZNE), Sigmund-Freud-Straße 27, 53127 Bonn, Germany

2 Institute of Complex Systems, Structural Biochemistry (ICS-6), Forschungszentrum Jülich GmbH, Wilhelm-Johnen-Straße, 52425 Jülich, Germany

3 Institut für Physikalische Biologie, Heinrich-Heine-Universität Düsseldorf, Universitätsstraße 1, 40225 Düsseldorf, Germany

Correspondence to: Gültekin Tamgüney,

Institute of Complex Systems, Structural Biochemistry (ICS-6), Forschungszentrum Jülich GmbH, Wilhelm-Johnen-Straße, 52425 Jülich, Germany

E-mail: erdem@fz-juelich.de

Telephone: +49-151-61324989

Fax: +49-2461-612023

Online Resource 2 Deposits of phosphorylated α-synuclein accumulate in the motor cortex of diseased TgM83$^{+/-}$ mice

Immunohistochemical staining with an antibody to α-synuclein phosphorylated at Ser129 revealed that diseased animals had accumulated deposits of phosphorylated α-synuclein in neuronal cell somata (arrow heads) and neurites (arrows) of the motor cortex (**a**). To assess pathology in the motor cortex in dependence on the route of inoculation, we separately counted the number of somata (**b**) and neurites (**c**) with α-synuclein pathology in each cortical layer (I, II-III, IV, V, VI). In the motor cortex, pathology was most pronounced after intracerebral inoculation and weakest after intraperitoneal inoculation, and intermediate after oral or intravenous challenge. Cell body pathology was absent in layer I and most pronounced in layer V. Neurites were sparsely present in layer I but frequent in all other layers. Data are presented as mean ± standard deviation and are derived from three to four animals per inoculation group. WM: white matter. Scale bars = 200 µm.

Online Resource 3 Deposits of phosphorylated α-synuclein colocalize with ubiquitin in the spinal cord of diseased TgM83$^{+/-}$ mice

Immunofluorescence staining of tissue sections of the spinal cord show that phosphorylated α-synuclein (red), detected with the EP1536Y antibody, and ubiquitin (green) colocalize in affected neurons of diseased mice. We observed similar results regardless of the route of challenge. Neurons of BSA-challenged mice, for which only merged images are shown, did not accumulate any excessively phosphorylated α-synuclein or ubiquitinated protein deposits. Nuclear staining with DAPI is shown in blue. Scale bar = 20 µm.

Online Resource 4 Deposits of phosphorylated α-synuclein colocalize with p62 in the spinal cord of diseased TgM83$^{+/–}$ mice

Immunofluorescence staining of tissue sections of the spinal cord show that phosphorylated α-synuclein (red), detected with the pSyn#64 antibody, and p62 (green) colocalize in affected neurons of diseased mice. We observed similar results regardless of the route of challenge. Neurons of BSA-challenged mice, for which only merged images are shown, did not accumulate any excessively phosphorylated α-synuclein or p62-tagged protein deposits. Nuclear staining with DAPI is shown in blue. Scale bar = 20 µm.

Online Resource 5 Astrogliosis in the spinal cord of diseased TgM83$^{+/–}$ mice

Immunofluorescence staining of spinal cord tissue sections with an antibody against GFAP, a marker for astrocytes, shows that neurons of diseased TgM83$^{+/–}$ mice with deposits of phosphorylated α-synuclein (red), which were detected with the pSyn#64 antibody, were frequently surrounded by reactive astrocytes (green). In contrast, no excessive abundance of phosphorylated α-synuclein or reactive astrocytes was observed in the spinal cord of BSA-challenged, healthy control mice. Nuclear staining with DAPI is shown in blue. Scale bar = 20 µm.

Online Resource 6 Microgliosis in the spinal cord of diseased TgM83$^{+/–}$ mice

Immunofluorescence staining of spinal cord tissue sections with an antibody against IBA-1, a marker for microglia, shows that neurons of diseased TgM83$^{+/–}$ mice with deposits of phosphorylated α-synuclein (red) , which were detected with the pSyn#64 antibody, were frequently surrounded by activated microglia (green) with an amoeboid morphology. In contrast, no excessive abundance of phosphorylated α-

synuclein or activated microglia was observed in the spinal cord of BSA-challenged, healthy control mice. Nuclear staining with DAPI is shown in blue. Scale bar = 20 μm.
